# Preliminary experience using MR-guided adaptive radiotherapy in head and neck cancer

**DOI:** 10.3389/fonc.2024.1474115

**Published:** 2024-11-08

**Authors:** Caiden Atienza, Andrew Shepard, Uwajachukwumma Uzomah, Shri Kiriti Rajan, Carryn M. Anderson, Joel Katzer, Samuel Rusu, Joel St-Aubin, Blake Smith, Daniel Hyer

**Affiliations:** University of Iowa Health Care, Department of Radiation Oncology, Iowa City, IA, United States

**Keywords:** adaptive, radiotherapy, benefit, MR-linac, organs-at-risk

## Abstract

This retrospective study evaluates the dosimetric benefits of adaptive radiotherapy for head and neck cancer patients. Five patients with node-positive oropharyngeal squamous cell carcinoma were treated with MR-guided radiotherapy using the Elekta Unity MR-Linac, undergoing 3-4 offline adaptive plan modifications during their treatment. This study compared the dose delivered to organs at risk (OARs) in a full offline adaptive approach versus an approach accounting only for daily setup. Results demonstrated a reduction in mean dose to critical structures in the offline adaptive arm. For example, the pharynx avoidance structure showed mean dose reductions ranging from 1.4 Gy to 3.6 Gy, and the left parotid gland exhibited reductions from 1.5 Gy to 1.9 Gy. Overall, offline adaptive radiotherapy reduced the mean cumulative dose to OARs in 19 of the 23 evaluated structures. Despite some instances of higher doses, the offline adaptive approach generally resulted in lower cumulative doses, emphasizing its potential to mitigate radiation-induced side effects. These findings suggest that offline adaptive radiotherapy has the potential to enhance treatment efficacy by better accommodating anatomical changes during therapy, thereby improving patient outcomes and reducing treatment-related morbidity.

## Introduction

Radiation therapy has long been founded on the premise that anatomical structures closely mimic their relative shape and location throughout the course of treatment (1). However, the advent of MR-guided Radiotherapy (MRgRT) challenges these assumptions (2, 3). Offering improved soft tissue contrast compared to traditional CT image-guided methods, MRgRT enables daily visualization of subtle, and potentially consequential, anatomical changes occurring over the 7-week long treatment for head and neck (H&N) cancer (4). This is especially evident in patients with HPV-associated oropharyngeal cancers (OPC) which commonly demonstrate a significant reduction in tumor size within the first weeks of treatment (5). As such, patients with HPV-associated OPC may be ideal candidates for adaptive treatment with MRgRT.

Our institution has embraced an adaptive workflow, empowering physicians to initiate offline plan adaptations on demand, ensuring treatment plans evolve in tandem with changing anatomy throughout the therapy course. This adaptive strategy holds considerable promise, particularly in the context of H&N cancer treatment, where tumors not only undergo substantial changes in size and shape throughout the course of treatment, but patients can also have significant weight loss due to side effects such as oral mucositis and subsequent dysphagia (6–9). The ability to adapt the plan to a shrinking tumor can mitigate radiation exposure to critical structures such as the pharyngeal constrictors, parotid and submandibular glands and holds significant clinical relevance (6, 10, 11), especially given the well-established association between radiation damage to these organs and the development of side effects such as xerostomia and late dysphagia (7, 8, 12). Due to the long course fractionation of these treatments, an offline adaptive strategy is appropriate since it reduces the on-table treatment time for head and neck patients, which is typically 30 minutes or less at our institution, while still achieving the benefits of adaptive therapy.

This study seeks to quantify the potential dosimetric benefits of offline adaptive planning in head and neck cancer using an in-silico analysis comparing two planning arms. Specifically, we aim to quantify the efficacy of an on-demand offline adaptive planning approach compared to a standard image guided approach that accounts only for patient setup error.

## Materials and methods

### Patient selection

Five patients were selected for this retrospective study. This human subjects research was reviewed and approved by the University of Iowa IRB-01 (Biomedical, application 201109821, Buatti principal investigator). Prospective consent for use of images was obtained for all participants; none of the elements were waived. All five patients selected for this study had node positive (N+) oropharyngeal squamous cell carcinoma and were treated with definitive chemoradiation therapy at the University of Iowa. All patients received a total of 7000 cGy in 35 fractions. Four of these patients were HPV positive (HPV+) and one was HPV negative (HPV-). All five patients received 70 Gy to areas of gross disease, including primary tumor and any grossly involved lymph nodes as determined by CT, MRI, PET/CT and physical exam. Elective nodal volumes spanned from 56-63 Gy, as determined by treating physician’s judgment of high or low risk of involvement.

All patients on this study were treated on the Elekta Unity (Elekta, Stockholm, Sweden) MR-Linac using MRgRT. They were initially selected for MRgRT as they represent a subset of patients where relatively larger changes in the tumor volume and patient anatomy would be expected, making them good candidates for offline adaptive replanning. Other patient-specific factors that informed selection for MRgRT included the size/extent of disease and proximity to critical structures, patient comorbidities, ability to tolerate prolonged treatment times, and patient preference. For the purpose of this retrospective study, these patients were selected because they had 3-4 offline adaptive plans over the course of their treatment (**Table 1**) which represents the median number of adaptations for the head and neck cancer cases treated on the MR-linac at our institution.

**Table 1:** Summary of patient diagnosis and treatment.

Patient	Diagnosis	Stage	Tumor Site	Volume of PTV7000 (cc)	# of Offline Adaptations	Fractions of Offline Adaptations
1	HPV+OPC	T2N1	Left Tonsil	237.8	3	2, 18, 32
2	HPV+OPC	T2N1	Left Base of Tongue	107.2	4	10, 14, 22, 25
3	HPV+OPC	T2N1	Left Base of Tongue	92.9	3	10, 12, 22
4	HPV-OPC	T2N3b	Right Tonsil	122.4	3	7, 11, 20
5	HPV+OPC	T1N1	Left Base of Tongue	153.1	3	14, 24, 34

### Treatment plan creation

All treatments were planned based on the standard clinical protocol for H&N treatments at the University of Iowa. Patients were simulated and had initial treatment planning using a primary reference CT for dose calculation. A 3T MR and PET-CT scan were also acquired and registered to the initial simulation CT to aid in target localization. The patients were immobilized in a H&N mask during simulation as well as subsequent treatments.

An initial reference plan was created for each patient based on the simulation imaging acquired prior to initiating treatment. The maximum number of IMRT segments in the step-and-shoot treatment plans ranged from 130 to 150. For each daily treatment, a pre-treatment T1-weighted MRI was acquired on the MR-Linac and registered with the CT reference image. Following initial registration, the adapt-to-position workflow (adapt shapes and weights) (13) was utilized to adapt the reference plan to account for the daily positional offset resulting from the three degrees of freedom rigid registration. In this workflow, there were no adjustments to target or OAR (organ-at-risk) contours for daily treatment. If during daily image review, the treating physician reviewed the plan and noted a clinically relevant change in target or OAR anatomy that warranted a change in contours, an offline adaptive plan using the ATS workflow would be ordered to account for the anatomical changes observed during treatment. The offline adaptive plan would be created prior to the next fraction using the daily MR image and would serve as the reference treatment plan for the ATP workflow in all subsequent fractions unless an additional offline adaptive plan was ordered. All offline adaptive replans were based on the clinical decision of the physician.

**Figure 1:**
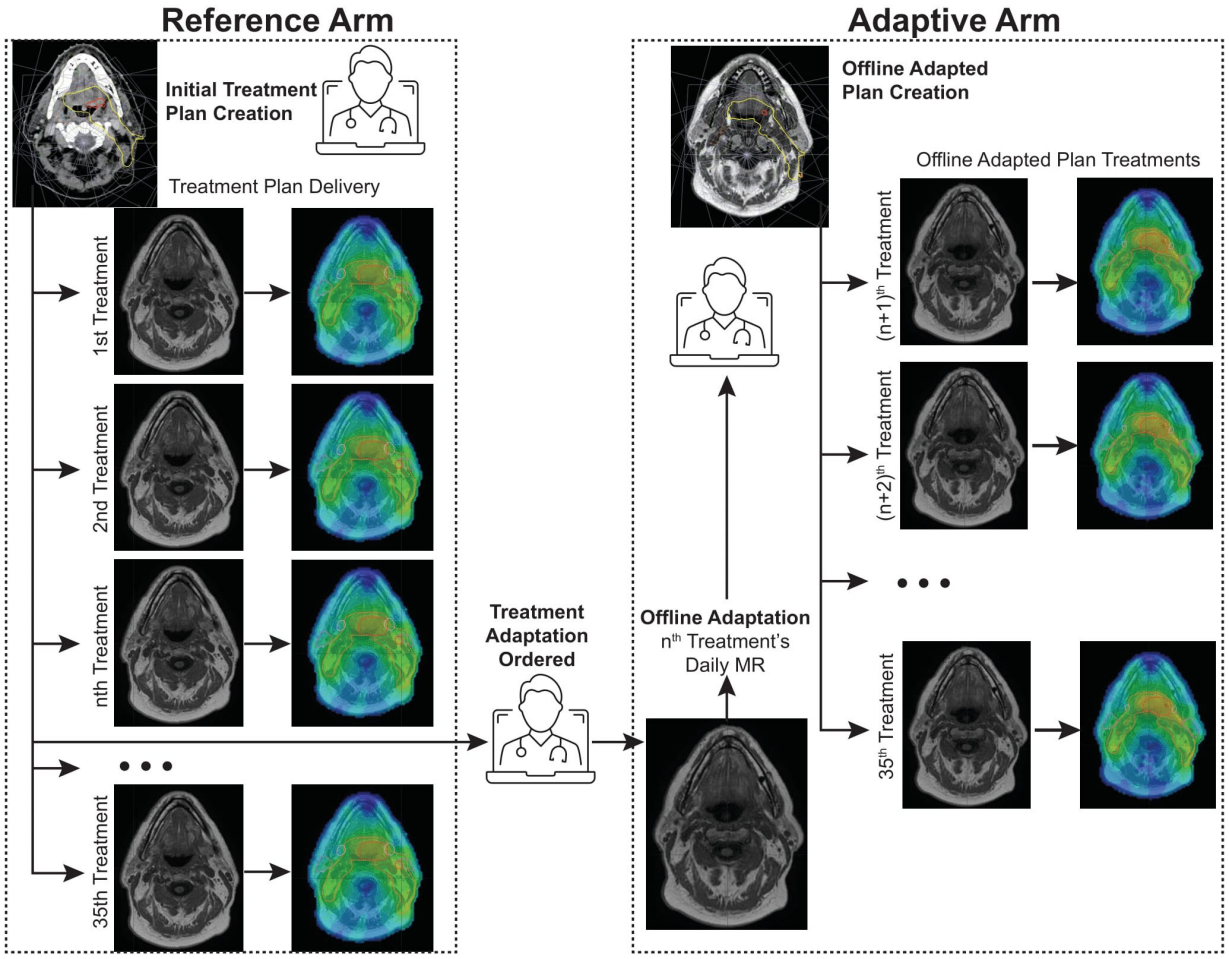
Reference Arm vs. Adaptive Arm workflows. The reference arm consists of using the original treatment plan as the reference for ATP planning of all treatment fractions while the adaptive arm is initiated after a treatment adaptation is ordered and is repeated each time the physician orders another treatment plan change.

For this study, a reference arm and an adaptive arm were used to evaluate the impact of this on-demand adaptive replanning approach. Each arm utilized re-contoured datasets and manual recalculation of the dose on the daily MR to accurately evaluate the delivered doses with anatomical changes that occur during treatment. Although deformable image registration (DIR) is employed during adaptations, all contours were manually verified and edited in this study. The target volumes were not changed in the daily fractions, except for the recontouring that occurred during offline adaptation. All doses on the daily MR were calculated using the Monaco treatment planning system (v6.2.1, Elekta) with a bulk density override strategy which assigns the electron densities to selected contours based on the mean density derived from the reference planning CT image (4). The calculated dose and structures were then exported to ProKnow for analysis. The reference arm simulates the scenario in which the original plan was used as the reference plan for the ATP workflow in all 35 fractions. In contrast, the adaptive arm simulated our clinical process for which offline ATS adaptations were made at the discretion of the physician. This offline adapted plan was then used as the reference plan within the ATP treatment workflow for the remaining fractions unless another adaptation was ordered, and the process was repeated. The offline adaptive arm therefore represents the clinically delivered plans with the additional step of recalculating each plan on the daily MR dataset as opposed to the reference dataset (as is typical for ATP planning). Plans in both arms were normalized to the same PTV7000 coverage for each fraction. To maintain a consistent comparison between the two arms, all plans relied on the clinical registrations performed and no modifications were made to adjust for a new reference plan. The two arms of the study are illustrated in **Figure 1**. Using this methodology resulted in 350 plans that were compared for this study.

### Dose evaluation

Five OARs were chosen for evaluation. The right and left parotid glands, the right and left submandibular glands, and a physician-created “pharynx avoid” structure. The “pharynx avoid” structure is a subjective OAR volume routinely used at our institution encompassing pharyngeal muscles spanning from C2 to the esophageal region vital to swallowing function. This structure is frequently optimized for a mean dose <40 Gy in the planning directive provided by the physician. Mean dose was chosen as the metric for analysis, as it is routinely evaluated by the physician during plan evaluation.

The cumulative mean dose for each OAR was calculated by summing the mean dose per fraction across all fractions. The mean dose per fraction was obtained by dividing the total reported dose by the number of fractions delivered, a method routinely used by physicians for plan assessment.

## Results

The cumulative mean doses to the OARs analyzed for the adaptive and reference arms are presented in **Table 2**. The offline adaptive arm exhibited a reduction in dose to the OARs surrounding the planning target volume (PTV) for 19 of the 23 organs evaluated. Two organs were not evaluated due to removal prior to treatment (left parotid for Patient 2) and/or being fully encompassed by the PTV (left submandibular for Patient 3). A cumulative mean dose savings was observed for the pharynx avoid structure in all five patients ranging from 1.4 Gy to 3.6 Gy. The left parotid also showed a cumulative mean dose savings for all four patients analyzed ranging from approximately 1.5 Gy to 1.9 Gy (**Table 2**). Offline plan adaptation reduced the mean dose to all OARs analyzed for 3 of the 5 patients.

**Table 2:** Summary of the cumulative mean doses for the OAR analyzed for each arm of the study.

	Cumulative Mean Dose (Gy)					
	Patient 1	Patient 2	Patient 3	Patient 4	Patient 5	Average Dose
Rt Parotid						
Reference	29.34	20.46	26.49	30.03	16.38	24.54
Offline	27.31	19.13	26.12	29.18	16.90	23.73
Difference, Gy(%)	-2.03 (-6.92)	-1.33 (-6.50)	-0.37 (-1.40)	-0.85 (-2.83)	0.52 (3.17)	-0.81 (-3.31)
Lt Parotid						
Reference	53.44	N/A	35.47	20.90	29.21	34.76
Offline	51.63	N/A	33.98	18.99	27.54	33.04
Difference, Gy(%)	-1.81 (-3.39)	N/A	-1.49 (-4.20)	-1.91 (-9.14)	-1.67 (-5.72)	-1.73 (-4.96)
Rt Submandibular						
Reference	46.32	61.07	62.33	58.78	37.60	53.22
Offline	44.14	61.00	60.87	60.24	39.48	53.15
Difference, Gy(%)	-2.18 (-4.94)	-0.07 (-0.11)	-1.46 (-2.40)	1.46 (2.42)	1.88 (4.76)	-0.07 (-0.14)
Lt Submandibular						
Reference	72.44	69.46	N/A	34.55	60.15	59.15
Offline	71.34	68.41	N/A	36.98	58.93	58.92
Difference, Gy(%)	-1.10 (-1.54)	-1.05 (-1.53)	N/A	2.43 (6.57)	-1.22 (-2.07)	-0.23 (-0.40)
Pharynx Avoid						
Reference	48.75	36.91	46.02	41.82	49.65	44.63
Offline	47.04	35.49	42.46	39.28	47.40	42.33
Difference, Gy(%)	-1.71 (-3.64)	-1.42 (-4.00)	-3.56 (-8.38)	-2.54 (-6.47)	-2.25 (-4.75)	-2.30 (-5.14)

Not applicable (N/A) denotes the organs which could not be analyzed due to removal or encompassed by the target.

There were four instances where the adapted arm delivered a higher dose to an OAR compared to the reference arm, specifically in patient 5’s right parotid gland and right submandibular glands, as well as both submandibular glands in patient 4. For 3 of the 4 instances, the doses to these OARs were relatively low compared to the average among all patients. The cumulative mean dose to the right parotid of patient 5 was 16.38 Gy for the reference arm, compared to the average cumulative mean excluding patient 5 of 28.39 Gy. Likewise, the right submandibular gland of patient 5 and left submandibular gland of patient 4 had a cumulative mean dose of 37.60 Gy and 34.55 Gy, respectively, compared to the average cumulative mean for the other patients’ submandibular glands which was much higher at 61.37 Gy.

**Figure 2:**
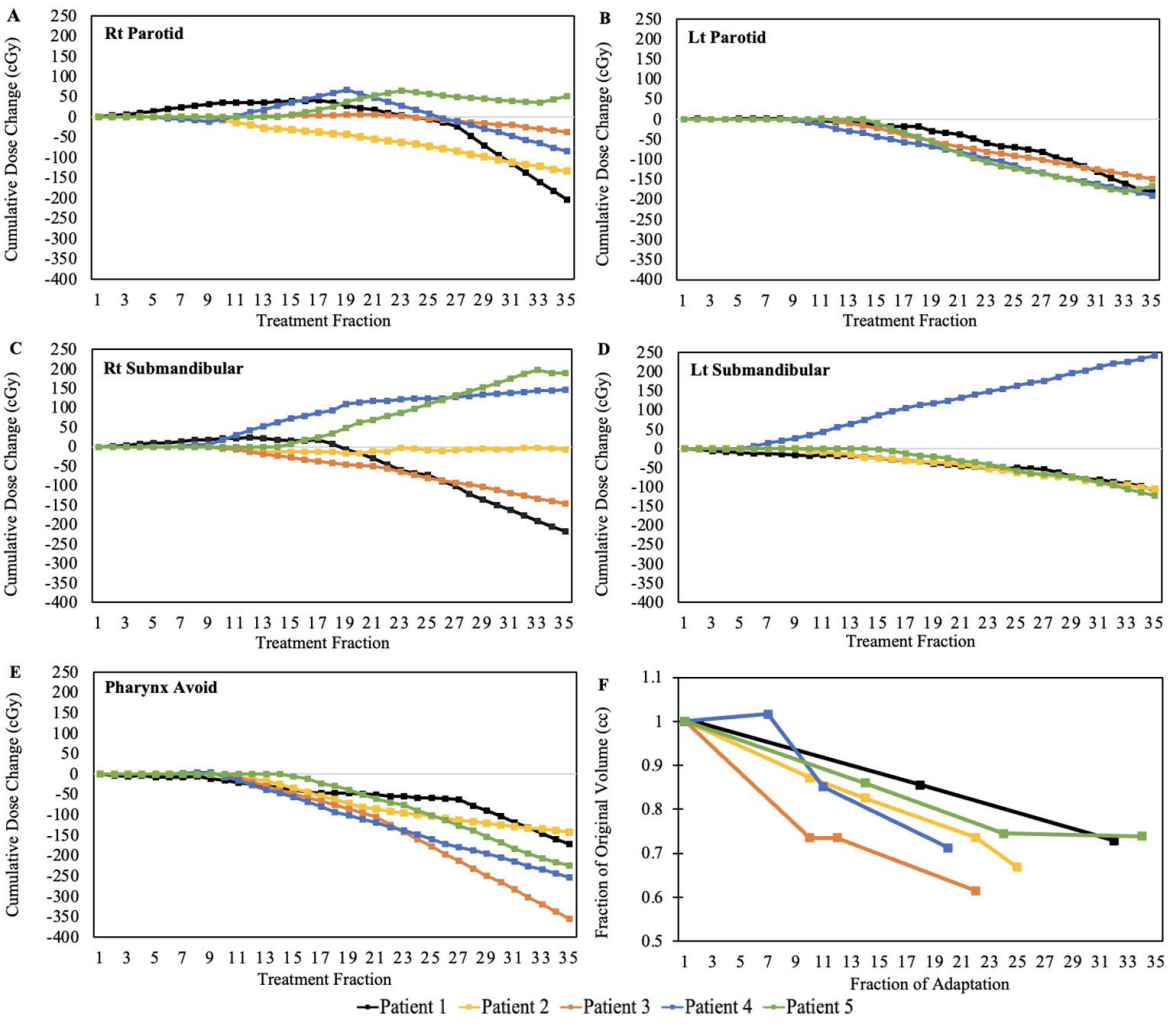
Cumulative dose change per fraction. Right Parotid **(A)**, Left Parotid **(B)**, Right Submandibular **(C)**, Left Submandibular **(D)**, Pharynx Avoid **(E)**, and high-dose target-volume reduction as a function of the fraction at which adaptation occurred **(F)**.

It is also important to point out the tumor volume changes throughout the course of treatment (**Figure 2F**). The gross tumor volumes for the five patients was on average reduced to approximately 65% of its original volume at the start of treatment, indicating the necessity of the offline replans.

## Discussion

Our results are comparable and consistent with a study done by the University of Zurich which evaluated the anatomical changes and cumulative dose to the salivary glands throughout the treatment course using two adaptation strategies (10). One strategy was a single adaptation after week 5 of treatment and the other was a weekly adaptation throughout the course of treatment. The study found significant anatomical changes and notable dose savings for majority of the salivary glands. The weekly adaptation strategy allowed for an average cumulative dose reduction of 5.4% to the parotids and 1.6% for the submandibular glands when compared to the single adaptation strategy. Our on-demand adaptation strategy for the patients analyzed in this study resulted in cumulative dose reductions of 4.1% for the parotids and 0.1% for the submandibular glands when averaged across all patients. The cumulative dose changes are illustrated in **Figures 2A–E**. While these values may appear minor, it is well known that parotid gland dysfunction is directly related to radiation dose, with the effects accelerating beyond 20 Gy (8). This dose relationship indicates that small margins of improvement, such as those presented in this work, may translate to improvements in function and quality of life for head and neck cancer patients.

In contrast to other works, our institution’s workflow uses physician discretion to trigger recontouring and replanning based on anatomical changes rather than following a routine adaptation schedule (10). By using the physician’s judgement to trigger the offline replan, our workflow avoids the additional time associated with on-table adaptations while also minimizing offline adaptations that may not be needed.

It is also crucial to address the cases/patients where dose savings were not necessarily observed following the offline adaptation. As indicated in the results, 3 of the 4 structures in which an increase was observed were receiving a relatively low dose in comparison to the same structures for other patients. As the dose was lower, less stringent optimization objectives were placed on these structures when performing offline adaptations in order to focus on other high priority structures initially receiving a higher dose. As the study was a retrospective investigation utilizing the clinical offline adaptive plans, the replanning strategy was not necessarily uniform across all cases. For example, the relative weight of optimization parameters and the tradeoff with other key structures was at the discretion of the dosimetrist and the attending physician for each case, with structures already receiving a low dose typically not being weighted as highly during optimization. The right submandibular gland of patient 4 also failed to exhibit dose savings despite a high cumulative mean dose of 58.78 Gy. When reviewing the optimization objectives, it was discovered this structure overlapped with the PTV and the desire to maintain PTV coverage superseded the goal of limiting the submandibular dose in the overlap region.

While this work presented promising results for offline adaptive planning in the scope of MRgRT, there were several limitations which include its retrospective nature, small sample size, and non-standard methodology for replanning. As with all retrospective studies, there is an open door for clinical bias through the patient selection process, where the cases selected may affect the validity of the conclusions drawn. To minimize this effect, we selected patients that were representative of our standard head and neck population treated with MRgRT and more specifically, patients that had a total number of adaptive replans that was representative of the median across all head and neck patients treated with MRgRT at our institution. An additional limitation to this study is that mean dose was summed rather than using a voxel-by-voxel dose summation technique. Summing the mean dose provides a conservative estimate to the total dose delivered without the uncertainty associated with voxel-by-voxel dose summation. Overall, the findings in this study are representative of our current clinical practice and can be helpful in drawing conclusions on the advantages of adaptive therapy in head and neck cancer.

## Conclusion

From the analysis of the cumulative mean dose of both the reference and adaptive arms for the patients analyzed, it was observed that offline adaptive radiotherapy led to a reduction in dose to OARs in most cases and presents a practical approach for improving patient treatments.

## Data Availability

The original contributions presented in the study are included in the article/supplementary material. Further inquiries can be directed to the corresponding author.
